# Hip Adductor Intramuscular Nerve Distribution Pattern of Children: A Guide for BTX-A Treatment to Muscle Spasticity in Cerebral Palsy

**DOI:** 10.3389/fneur.2019.00616

**Published:** 2019-06-14

**Authors:** Yan Yan, Xiaoyun Fu, Xiadan Xie, Songling Ji, Huaixiang Luo, Fangjiu Yang, Xiaoming Zhang, Shengbo Yang, Peng Xie

**Affiliations:** ^1^Department of Anatomy, Zunyi Medical University, Zunyi, China; ^2^Department of Critical Care Medicine of the Third Affiliated Hospital, Zunyi Medical University, Zunyi, China; ^3^Department of Critical Care Medicine of the Affiliated Hospital, Zunyi Medical University, Zunyi, China; ^4^Department of Biochemistry, Zunyi Medical University, Zunyi, China; ^5^Department of Molecular and Cellular Biology, Baylor College of Medicine, Houston, TX, United States

**Keywords:** hip adductor, intramuscular nerve, cerebral palsy, spasticity, botulinum toxin type A (BTX-A)

## Abstract

To investigate the intramuscular nerve distribution pattern in the hip adductors of children and to precisely locate the injection site for botulinum toxin type A (BTX-A) as a treatment for hip adductor spasticity in children with cerebral palsy. Modified Sihler's whole mount nerve staining technique was employed to observe the distribution of intramuscular nerves in hip adductors of children and to further locate zones where terminal nerves are concentrated. The terminal nerves of the adductor longus appeared in a longitudinal distribution band parallel to the line between the upper 1/3 point of the lateral boundary and the center of the medial boundary. In adductor brevis, the terminal nerves showed a sheet-like distribution with a nerve dense area located in the middle of the muscle belly that extends from the upper-inner region to the lower-outer region. Gracilis showed a dense area of terminal nerves in the middle of the muscle belly, closer to the posterior boundary. In adductor magnus, the dense area of terminal nerves showed a sheet-like distribution in the middle and lower region of the muscle belly. The dense area of terminal nerves in the pectineus was located in the middle of the muscle belly. This study is the first to systematically investigate the intramuscular nerve distribution pattern in the hip adductors. The results indicated that the best targets for BTX-A injection, when treating spasticity, are the dense regions of terminal nerves described above.

## Introduction

Cerebral palsy (CP) is a syndrome caused by non-progressive brain injuries and developmental defects during pregnancy and infancy. The primary manifestations of this syndrome are dyskinesia and abnormal posture (1). Recently, with developments in perinatal medicine, the diagnosis of cerebral palsy has been increasing. The incidence of the disease among live births is 2.11–3.1% (1–3). The most common type of cerebral palsy is spastic cerebral palsy, which comprises ~78–80% of all cases (4, 5). Spasticity is the primary factor that not only causes disabilities in children, but also affects their psychological and physical development (6). Therefore, an effective treatment for muscle spasticity caused by cerebral palsy is a pressing issue.

Since the first use of botulinum toxin type A (BTX-A) for the treatment of muscle spasticity caused by cerebral palsy (7), many studies have supported the use of BTX-A as an ideal treatment for this problem (8–12). BTX-A can selectively function at the neuromuscular junction, i.e., the motor end-plate. It can suppress the release of acetylcholine without influencing its synthesis or storage. However, due to the dose-dependent properties of BTX-A, the effectiveness of treatment depends on the total dose delivered to the neuromuscular junction where the terminal nerves inside the muscles are found (13). If the injection site is inappropriate, reduced muscle tension may not be achieved, and side effects such as over-relaxation may occur. Additionally, because of the high cost of BTX-A, increasing the injection dose can be an additional economic burden for the families of children undergoing treatment. Although the current techniques help with locating the injection site, such as ultrasound, electromyogram, and electrical stimulation, can assist the operator, the injection target may be difficult to confirm due to smaller muscle sizes in children and lack of patient cooperation (14). Therefore, a precise injection location in the affected muscle is the key to an effective treatment of muscle spasticity using BTX-A in patients with cerebral palsy.

Hip adductor spasticity is a common symptom in children with cerebral palsy (15–17). Chronic or repetitive spasticity can result in changes in the rheological properties of the muscles. The spastic, stiff, and fibrotic hip adductor muscles can create pathological mechanisms and delay lower body motion development (18). Moreover, the spasticity of hip adductors can cause a progressive subluxation of the hip joint and lead to joint dislocation (19–22). There has not been a systematic study on the distribution of intramuscular nerve pattern in the hip adductors. Current literature cannot guide clinical practice to carry out accurate injection of BTX-A as a treatment for hip adductor spasticity. Although there have been reports about the intramuscular nerve distribution in adductor longus and the gracilis muscles, these studies were performed on adult muscles, not that of children. In addition, only the distributions of larger nerve bundles were reported (23, 24).

The modified Sihler's whole mount nerve staining technique is an internationally acknowledged method for tracing the intramuscular nerves without damaging muscle integrity (25–27). The technique allows a clear three-dimensional display of terminal nerves (i.e., the site where an α-motor nerve ending attaches to the muscular fiber, namely, the motor end-plate) that cannot be observed by naked eyes. Therefore, it has become an important method for precisely locating targets for BTX-A injection (28, 29). Recently, we exploited this technique to illustrate the distribution of intramuscular nerve endings in the trapezius muscle. The study provided precise locations for the medicinal treatments of chronic neck pain and yielded successful treatment outcomes (30).

In this study, the modified Sihler's technique was used to treat hip adductor muscles. The dense intramuscular terminal nerve regions were investigated. We aim to provide more precise anatomical locations for BTX-A injection and to improve the treatment of hip adductor spasticity in children with cerebral palsy.

## Materials and Methods

This study was approved by the Institutional Review Board (Ethical Application Ref: LS20162-021). The cadavers were donated with the consent of the families. There were 26 formaldehyde-fixed pediatric cadavers of 11 males and 15 females ranging from age 3–11 with an average age of 6.5. There were no apparent pathology, such as trauma, histories of surgical operations, or anatomical abnormalities in the hip adductors, detected from the lower limbs among the cadavers.

### Gross Anatomy Observation

The cadavers were placed in supine position. The skin and superficial fascia were separated layer by layer. The hip adductor muscles (adductor longus, adductor brevis, gracilis, adductor magnus, and pectineus) were clearly exposed. The locations of the extramuscular nerves and the nerve entry points to the muscles were carefully observed in all 26 cadavers (52 limbs). The distance between the primary extramuscular nerve bundle and the nerve entry point was measured for each muscle.

### Staining of the Intramuscular Nerves of the Hip Adductor

The modified Sihler's whole mount nerve staining technique was employed for muscle staining. The procedure is described below. Depigmentation: Muscle specimens were rinsed for 12 h under running water and then immersed in 3% KOH solution (adding 0.4 mL of 3% H_2_O_2_ for every 100 mL). The immersing solution was replaced when it turned cloudy. The specimens were removed from the solution after turning soft and translucent. This process lasted ~4 weeks. The specimens were rinsed under running water for 30 min after depigmentation and then soaked in Sihler's solution I (1:2:12 glacial acetic acid:glycerin:1% aqueous chloral hydrate) for decalcification. The solution was replaced every 3 days. The muscle specimens became more translucent after 3 weeks. After decalcification, the specimens were rinsed under running water for 30 min and placed into Sihler's solution II (1:2:12 Ehrlich's hematoxylin: glycerin: 1% w/v aqueous chloral hydrate) for staining. This process took ~2–3 weeks. The stained specimens were then placed into solution I for depigmentation. The contrast between the muscle and nerve fibers was examined using a light box till purple nerve branches and light blue muscle fibers were observed. This process took ~4–8 h. The depigmented muscle specimens were soaked in deionized water for 30 min followed by 1 h soaking in a 0.05% lithium carbonate solution; this step resulted in the color change of the nerve fibers from dark purple to purple black.

The muscle blocks were placed in a gradient of 40, 60, 80, and 100% glycerin until translucent; the processing at each step of the gradient took ~3 weeks. The distribution of the nerve branches was clearly visible after this process. The entire treatment process using the glycerin gradient lasted ~4 months. The muscle specimens were laid flat on a light box. The distributions of the terminal nerve branches in the muscles were carefully observed and recorded. Photographs and drawings were made.

### Statistical Analysis

All experimental data were analyzed using SPSS 17.0. The significance level, α, was set at 0.05. Quantitative data are expressed as the mean ± standard deviation. The differences between the two sides of each cadaver were analyzed by a Wilcoxon signed-rank test.

## Results

### Intramuscular Nerve Distribution Pattern of the Adductor Longus

**Figure 1:**
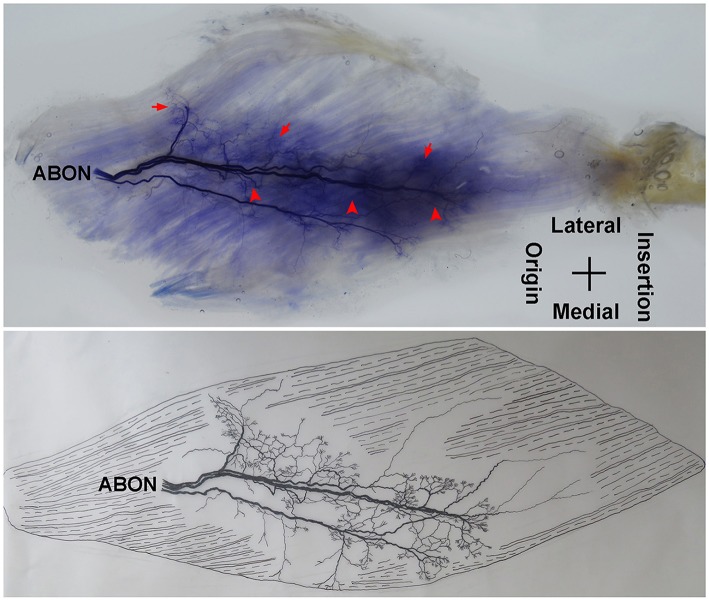
Distribution of intramuscular nerves in the adductor longus (right, deep side). The arrow indicates the tree-branch-like terminal nerves, and the arrowhead indicates the grid-like terminal nerve junctions (ABON, anterior branch of the obturator nerve).

**Figure 2:**
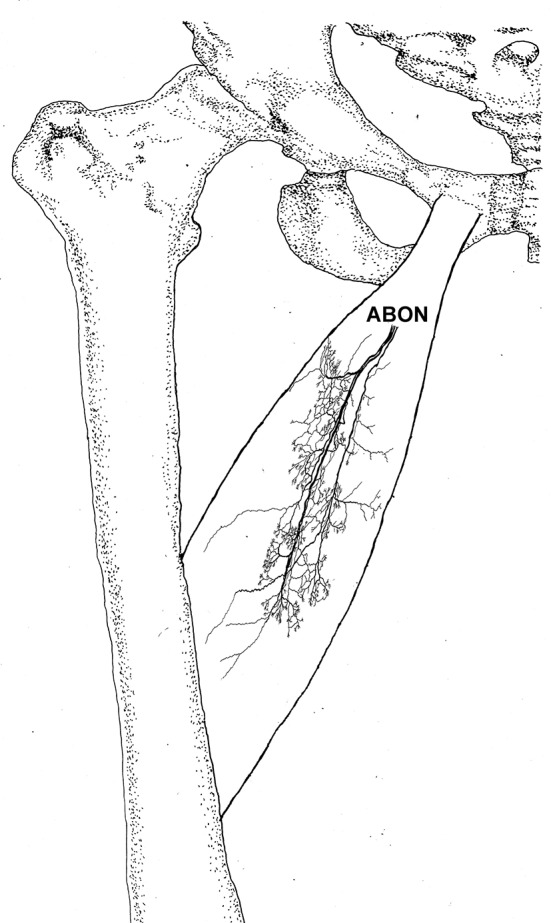
Three dimensional pattern of intramuscular nerve distribution in the adductor longus (right, deep side; ABON, anterior branch of the obturator nerve).

The adductor longus is innervated by the anterior branch of the obturator nerve. This branch is distributed between the adductor longus and the adductor brevis. The average length of this branch is 3.56 ± 0.4 cm. It travels along the deep surface of the adductor longus and enters the muscle at ~the 1/3 point on the muscle belly. After entering the muscle, the nerve splits into two primary medial and lateral branches. The medial branch is thinner comparing with the lateral branch. Both branches show a tree-branch-like distribution in the middle section of the muscle on medial side. The medial branch spreads to ~the 1/3 point and is distributed toward the center of the medial boundary. The branch is at a 45° angle with the muscle fibers and stops at the midpoint of the medial boundary, and there are many terminal nerve branches along the medial branch (Figures 1, 2).

The distribution of the lateral branch is similar to that of the medial branch and it stops at the midpoint of the medial boundary. After entering the muscle, the lateral branch splits into two secondary nerve branches. One of the secondary branches travels toward the lateral boundary of the adductor longus. There are various small branches reaching muscle fibers along with their distribution. The lateral branch terminates at the lateral boundary. Another branch spreads with the medial branch toward the medial boundary of the adductor longus. There are also various small branches reaching muscle fibers along its distribution. The terminal branches extend from the lateral and medial branches and form grid-like junctions. The junctions are loosely distributed in the top half and more saturate toward the midpoint of the medial boundary. The terminal branches of the adductor longus show a long band distribution. The densest region of terminal branches is located along the line formed between the upper 1/3 point of the lateral boundary and the center of the medial boundary (Figures 1, 2).

### Intramuscular Nerve Distribution Pattern of the Adductor Brevis

**Figure 3:**
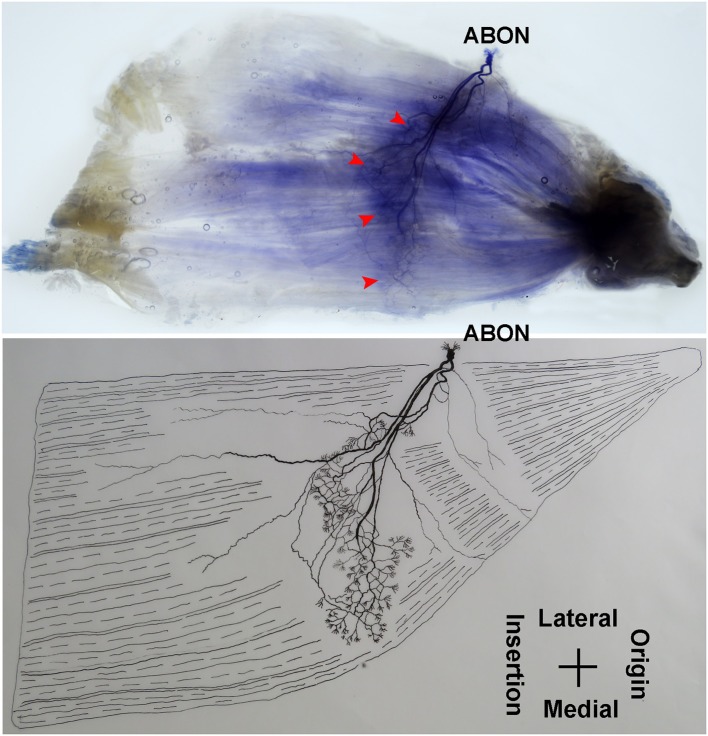
Distribution of intramuscular nerves in the adductor brevis (right, superficial side). The arrowhead indicates the grid-like terminal nerve junctions (ABON, anterior branch of the obturator nerve).

**Figure 4:**
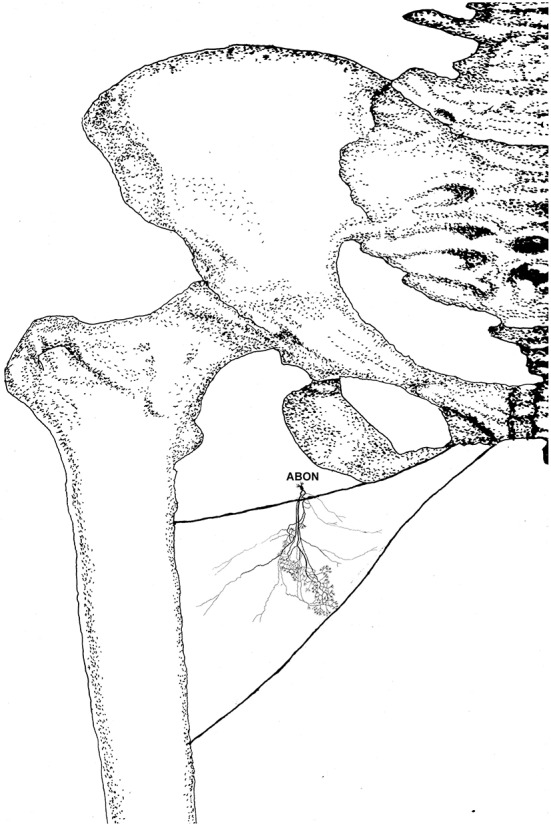
Three dimensional pattern of intramuscular nerve distribution in the adductor brevis (right, superficial side; ABON, anterior branch of the obturator nerve).

The anterior branch of the obturator nerve enters the front of the middle to lateral boundary of the adductor brevis. The extramuscular nerve trunk splits into two primary branches, the medial and lateral branches. The average length of the medial and lateral branches are 2.32 ± 0.3 and 2.89 ± 0.5 cm, respectively. The lateral branch splits into two secondary branches, which travel perpendicular to the muscle fibers. Along its spread, there are 4–5 smaller branches that terminate at the medial boundary. Additionally, there are 2–3 fine nerve fibers extend from the lateral branch and control the muscle fibers at the end of the adductor brevis. Thin branches are extend from the medial branch control the muscle fibers at the origin and end of the adductor brevis. The medial and lateral branches form a large number of grid-like terminal nerve junctions in the middle of the muscle belly. The terminal branches of the adductor brevis show a sheet-like distribution. The densest area of terminal branches is located at the center of the muscle belly and extend from the upper-medial region to the lower-lateral region (Figures 3, 4).

### Intramuscular Nerves Distribution Pattern of the Adductor Magnus

**Figure 5:**
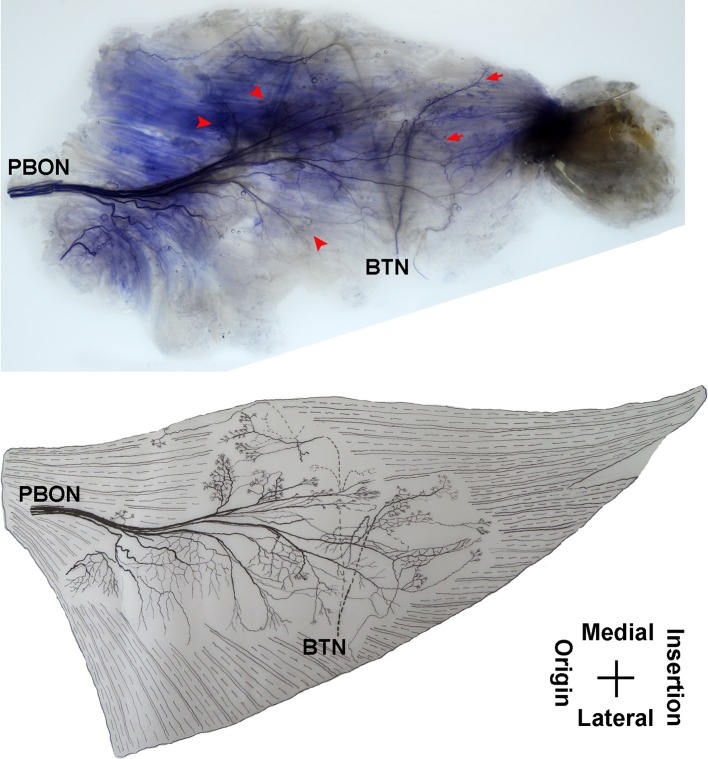
Distribution of intramuscular nerves in the adductor magnus (right, superficial side). The arrow indicates the O-shaped and Y-shaped terminal nerve junctions, and the arrowhead indicates the claw-like terminal nerve distribution (PBON, posterior branch of the obturator nerve; BTN, branch of the tibial nerve).

**Figure 6:**
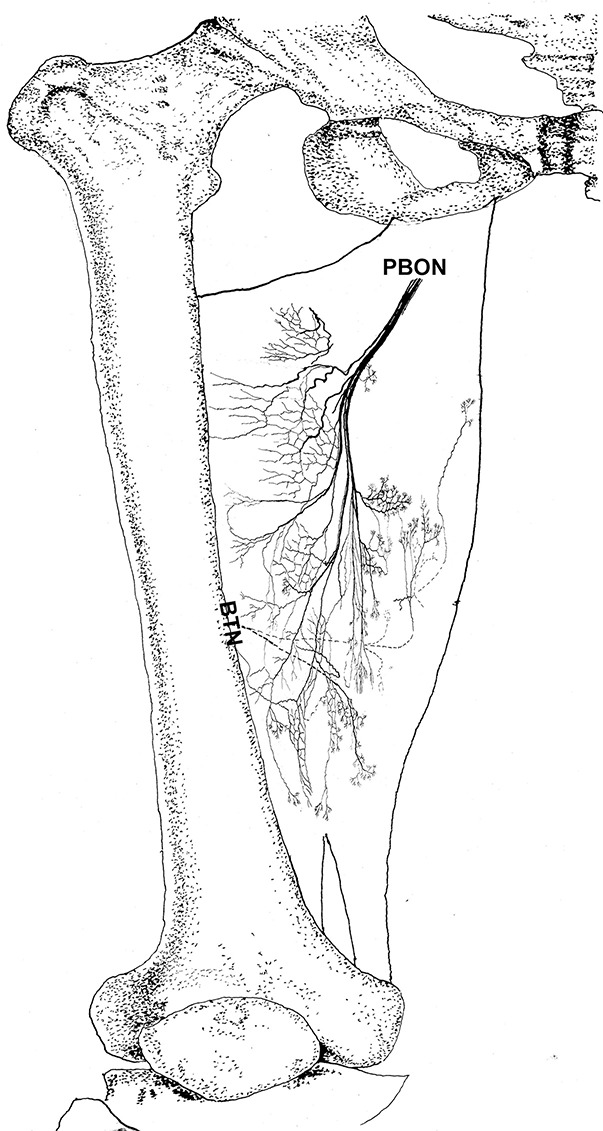
Three dimensional pattern of intramuscular nerve distribution in the adductor magnus (right, superficial side; PBON, posterior branch of the obturator nerve; BTN, branch of the tibial nerve).

The adductor magnus is innervated by both the posterior branch of the obturator nerve and a branch of the tibial nerve. The posterior branch of the obturator nerve is distributed between the adductor brevis and the adductor magnus. The average length of the extramuscular nerve trunk is 1.65 ± 0.2 cm. This nerve has a larger diameter and enters the muscle at the upper 1/3 point of the surface layer. The primary branch of the nerve extends toward the adductor muscular tendon after entering the muscle. Four to five fan-like branches extend from the primary branch toward the muscle fibers near the crista femoris and the terminal branches exhibit a dendritic shape. Additionally, the posterior branch of the obturator nerve divides into two 2–3 branches at the upper adductor magnus to innervate the medial muscle fibers. The terminal nerves show a claw-like distribution (Figures 5, 6).

The branch of the tibial nerve enters the adductor magnus from the center of the crista femoris of the adductor magnus. The average length of the branch is 2.04 ± 0.4 cm. After entering the muscle, this branch split into upper, middle, and lower branches. The upper branch extends along the medial boundary of the adductor magnus and at a 105° angle with the muscle fibers. Which splits into three thinner branches to innervate the muscle fibers at the center of the adductor magnus. The middle and lower branches each extend 3–4 fine nerve fibers that control the skeletal muscles near the end of the adductor magnus. O-shaped and Y-shaped junctions were observed the two branches (Figures 5, 6).

The intramuscular nerves of the adductor magnus show certain distinct characteristics. A few grid-like junctions formed by branches of the obturator nerve, which innervate the muscle fibers near the crista femoris. The branch of tibial nerve that controls the muscle fibers at the medial boundary also forms some linear junctions with the obturator nerve branch and innervate the muscle at the medial boundary. The obturator nerve branch, which innervate the muscle near the tendon of adductor magnus, form junctions with the middle and lower branches of tibial nerve. Thus, the densest region of terminal nerves in the adductor magnus show a sheet-like distribution in the middle to lower regions of the muscle belly (Figures 5, 6).

### Intramuscular Nerve Distribution Pattern of the Gracilis

**Figure 7:**
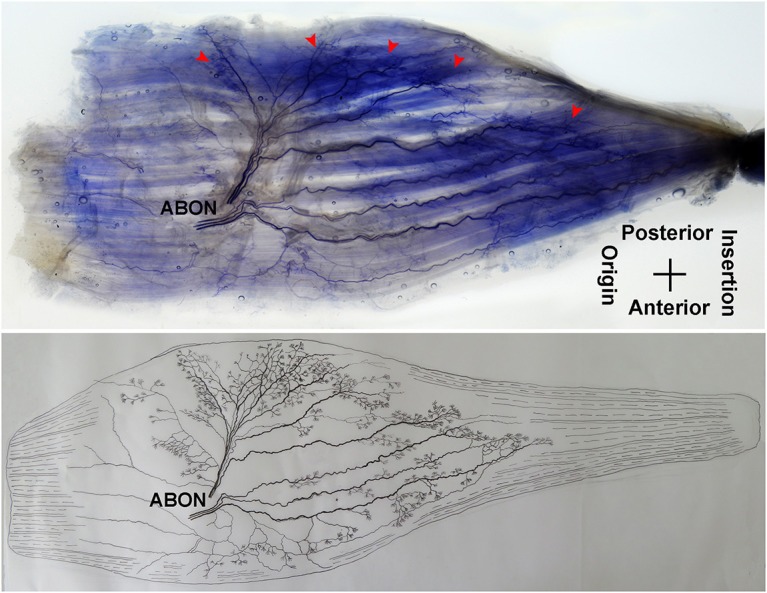
Distribution of intramuscular nerves in the gracilis (left, deep side). The arrowhead indicates the claw-like and tree-like terminal nerve distribution (ABON, anterior branch of the obturator nerve).

**Figure 8:**
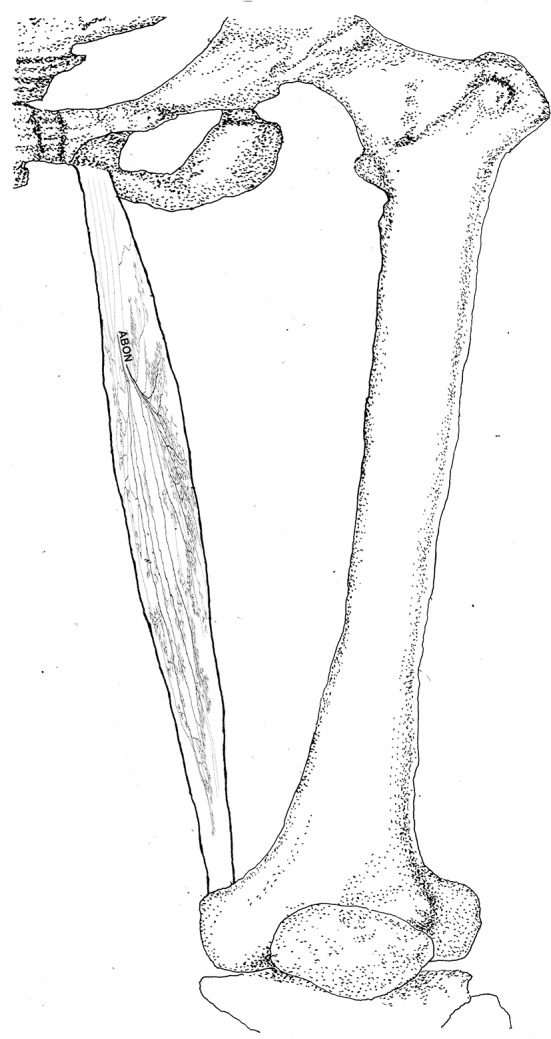
Three dimensional pattern of intramuscular nerve distribution in the gracilis (left, deep side; ABON, anterior branch of the obturator nerve).

The anterior branch of the obturator nerve spread between the adductor longus and the adductor brevis. This branch starts from the medial boundary of the adductor longus and penetrates the gracilis. The branch splits into two primary branches before entering the muscle. The average length of the two primary branches are 1.87 ± 0.2 and 2.16 ± 0.4 cm, respectively. One of the branches travels along the muscle fibers and splits into 4–5 small branches, and then extend radially toward the origin and end of the muscle as well as toward the anterior boundary. The small branches extend toward the end of the muscle and spread into claw-like terminal branches. The other primary branch extends into 8–9 fine branches in a fan-like distribution at the center of the muscle, toward the posterior boundary. Three to four of the fine branches spread toward the origin of the muscle, while the others extend toward the end of the muscle. These branches extend into a large number of claw-like and tree-like terminal nerves which reach the muscle fibers at the posterior of the gracilis (Figures 7, 8).

The distribution of intramuscular nerves in the gracilis show a few distinctive characteristics. The terminal branches reaching the anterior muscle belly don't show apparent nerve junctions, while there are a large number of nerve fibers near the center of the posterior muscle belly. The terminal branches also form grid-like junctions. Therefore, the densest region of terminal branches is located at the center of the muscle belly, toward the posterior boundary (Figures 7, 8).

### Intramuscular Nerves Distribution Pattern of the Pectineus

**Figure 9:**
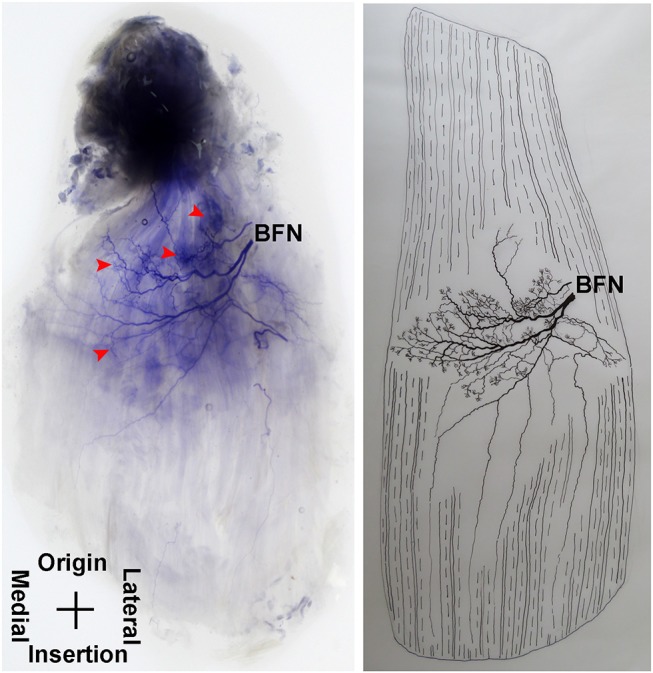
Distribution of intramuscular nerves in the pectineus (right, superficial side). The arrowhead indicates the tree-like terminal nerve distribution (BFN, branch of the femoral nerve).

**Figure 10:**
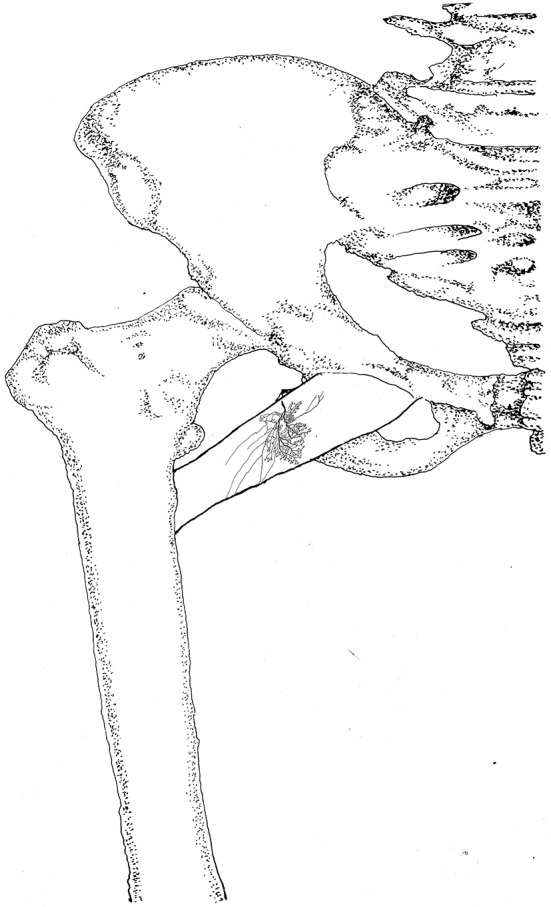
Three dimensional pattern of intramuscular nerve distribution in the pectineus (right, superficial side; BFN, branch of the femoral nerve).

The pectineus is a short muscle with a rectangular shape and is innervated by the branch of the femoral nerve. The average length of the extramuscular nerve trunk is 1.96 ± 0.3 cm. The femoral nerve enters the muscle from the middle of the lateral boundary of the surface layer of the pectineus. After entering the muscle, the nerve splits into an upper and a lower primary branches. The upper primary branch is thinner and extends toward the medial boundary. It splits into 3–4 branches upon reaching the center of the muscle belly. These branches further spread into claw-like terminal nerves and form Y- and O-shaped junctions. Additionally, a fine branch extends from the root of the primary branch toward the origin of the muscle. Five to six secondary branches extend along the primary branch and innervate the muscle fibers at the origin of the muscle. The lower primary branch extends perpendicular to the direction of the muscle fibers and spread along the upper primary branch toward the medial boundary of the muscle. Along its spread are many claw-like terminal nerves that control the nearby muscle fibers. There are a few linear junctions between the terminal branches of the two primary nerves. Based on the distribution of the intramuscular nerves on the pectineus, the densest region of terminal nerves is located at the center of the muscle belly (Figures 9, 10).

## Discussion

The treatments for muscle spasticity in children with cerebral palsy include physical therapy, acupuncture, oral administration of muscle relaxants, intrathecal injection of baclofen, local injection of alcohol and BTX-A, and surgical intervention (31–37). Children with cerebral palsy are a special group. The clinical practices of physical therapy and acupuncture often cause fear among children. Treatments such as the oral administration of medicine and the intrathecal injection of baclofen are often accompanied by undesired side effects including lack of muscle strength, loss of sensation, and hypersomnia. Local injection of alcohol and surgical processes can cause tissue damage. They can also trigger pain and permanent loss of nerve function. These treatments may not be suitable for early stages of muscle spasticity in children.

Studies have suggested that BTX-A is a safe and effective medication for treating muscle spasticity in children with cerebral palsy (8, 11, 38). It functions at the motor end plate, reduces muscle contraction, and re-establishes the balance between the primary motor muscle and the antagonistic muscle. These effects result in symptom relief, posture adjustment, and motor functions improvement. Additionally, BTX-A does not cause damage to the synaptic structure. It temporarily causes nerves to lose control of skeletal muscle. The functional connection, nerve conduction, and muscular activities are gradually restored, taking between a few weeks and 7 months (27, 39). Therefore, the anti-spasticity function of BTX-A is reversible. However, when treating spasticity, in order to reduce the necessary dose of BTX-A and to allow its maximum effects, the most suitable injection site must be determined. Therefore, a systematic study of the terminal nerves in the hip adductors is required.

There has been a study on the nerve entry points (motor points) of the hip adductor muscles. The motor points were confirmed by the line from pubic symphysis to medial condyle of the femur and possible injection targets for BTX-A were located based on this study (40). However, the nerve entry points are not equivalent to dense regions of terminal nerves. There is a certain distance between the two (29). Moreover, some researchers applied barium sulfate to the anterior and posterior branches of the obturator nerve as well as to the surface of the muscles in order to observe the distribution of the extramuscular nerves under x-ray, and then to measure the location of the blocking targets of the extramuscular nerves of the hip adductors (41). Although the above-described studies provided information for nerve block in the hip adductors, there was no mentioning of the intramuscular nerve distribution and the location of terminal nerve dense areas. Therefore, these studies did not provide an ideal guidance for BTX-A injection treatment for hip adductor spasticity in patients with cerebral palsy.

In our study, we systematically investigated the distribution of intramuscular nerves and dense areas of terminal nerves in the hip adductors of children. The results indicated that the terminal nerves of the adductor longus showed a long band of distribution along the line between the upper 1/3 point of the external boundary and the midpoint of the medial boundary. Won et al. applied this staining technique to observe the distribution of intramuscular nerves in the adductor longus of adults and found that the intramuscular nerves in the adductor longus tend to be distributed at the proximal, intermediate, and distal regions of the muscle. Among these locations, the terminal nerves were the most dense in the intermediate region (24). The results of their study were different from those observed in our study. These differences may be attributed to the smaller muscles and the larger dense area of nerves in children. Therefore, the best site for BTX-A injection in children experiencing adductor longus spasticity should be between the upper 1/3 point on the external boundary and the midpoint of the medial boundary.

There have been no reports on the distribution of intramuscular nerves in the adductor brevis. Our study found that the medial and lateral branches extended from the anterior branch of the obturator nerve and innervated the adductor brevis. A large number of junctions were observed between the medial and lateral branches and the branches were distributed in a sheet-like manner. Based on the distribution of the intramuscular nerves, the dense region of nerves in the adductor brevis was located at the center of the muscle belly (the sheet that extends from the upper-medial region to the lower-lateral region). Therefore, the injection site for BTX-A as a treatment for spasticity should be at the center of the muscle belly.

Won et al. utilized Sihler's staining to perform studies on the gracilis of adults. The results indicated that the dense region of terminal nerves in the gracilis was in a narrow zone at the center of the muscle belly (~29.2–33.5% from the anterior superior iliac spine) (24). However, our study found that the dense region of terminal nerves was located at the mid-muscle belly region, closer to the posterior boundary. This finding was different from that of Won et al. The difference may be attributed to the subjects in the studies; child cadavers were the subjects of our study. Therefore, for the treatment of gracilis spasticity, the ideal injection site for BTX-A is the mid-muscle belly region near the posterior boundary.

Until now, there have been no reports of histochemical staining studies on the intramuscular nerves of the adductor magnus and the pectineus. Studies have observed the neural control in the adductor magnus by autopsy (40, 42). The use of autopsy studies for use in guiding BTX-A injection may be limited because the locations of the neuromuscular junction (the dense region of terminal nerves) cannot be observed. The results of our study showed that the adductor magnus was innervated by both a tibial nerve branch and an obturator nerve posterior branch. There were grid-like and linear junctions between the terminal nerves. The dense region of terminal nerves was observed to have a sheet-like distribution and was located at the middle to lower sections of the adductor magnus. Therefore, the middle to lower sections of the muscle should be the BTX-A injection site. Additionally, our study clearly displayed the distribution of intramuscular nerve branches in the pectineus. The dense region of terminal nerves was located at the center of the muscle belly, which is the recommended BTX-A injection site.

This study also has some limitations. The number of individuals with different age was small. The dimensional distortion was done in some cases and the counting of twigs is rather ambiguous in Sihler's staining. In the future, a larger individuals with different age will be used for assessing intramuscular nerve distribution pattern, and we will continue to improve Sihler's staining technology to clearly show the terminal nerves.

## Conclusions

This study is the first to systematically investigate the intramuscular nerve distribution pattern in the hip adductors. When treating hip adductor spasticity in children with cerebral palsy, the ideal BTX-A injection site should be at the dense regions of terminal nerves in each muscle, which are described below. For the adductor longus, the site should be between the upper 1/3 point on the lateral boundary and the center of the medial boundary. For the adductor brevis, the site should be the sheet-like region at the center of the muscle belly that extends from the upper-medial area toward the lower-lateral area. For the gracilis muscle, the site should be at the center of the muscle belly, closer to the posterior boundary. For the adductor magnus, the site should be the middle and lower portion of the muscle belly. For the pectineus, the site should be the center of the muscle belly. Injections of BTX-A into the dense regions of terminal nerves described above will allow the maximum treatment effects while minimizing side effects. Therefore, muscle spasticity can be relieved, which will further improve lower limb function.

## Ethics Statement

This study was approved by the Institutional Review Board (IRB). The cadavers were donated with the consent of the families.

## Author Contributions

PX conceived and designed the experiments. YY, XF, XX, and PX performed the experiments. SJ, HL, FY, XZ, and SY analyzed the data. PX and XZ wrote the paper.

### Conflict of Interest Statement

The authors declare that the research was conducted in the absence of any commercial or financial relationships that could be construed as a potential conflict of interest.
